# Effects of Biphenyldimethyl-dicarboxylate Administration Alone or Combined with Silymarin in the CCL_4_ Model of Liver Fibrosis in Rats

**DOI:** 10.1100/tsw.2007.193

**Published:** 2007-08-24

**Authors:** Omar M. E. Abdel-Salam, Amany A. Sleem, Fatma A. Morsy

**Affiliations:** ^1^ Pharmacology, National Research Centre, Tahrir St., Dokki, Cairo, Egypt; ^2^ Pathology, National Research Centre, Tahrir St., Dokki, Cairo, Egypt

**Keywords:** biphenyldimethyldicarboxylate, silymarin, carbon tetrachloride, liver, rat

## Abstract

The effect of biphenyldimethyldicarboxylate (DDB), a synthetic compound, in use for the treatment of chronic hepatitis was studied on hepatic injury caused in rats by administration of carbon tetrachloride (CCl_4_). Starting at time of administration of the first dose of CCl_4_, rats received DDB at four dose levels (3, 15, 75 or 375 mg/kg), silymarin (22 mg/kg), a combination of DDB (75 mg/kg) and silymarin (22 mg/kg) or saline (control) once orally daily for 30 days. The administration of DDB in CCl_4_-treated rats at 75 or 375 mg/kg resulted in 61.2-76.2% decrease in alanine aminotransferase (ALT) and 46.9-60.8% decrease in aspartate aminotransferase (AST), respectively compared with the CCl_4_ control group. Silymarin treatment resulted in 34.6 and 30% decrease in ALT and AST, while DDB (75 mg/kg) combined with silymarin (22 mg/kg) resulted in 58.2 and 31% decrease in ALT and AST, respectively. Serum creatinine increased by 50% by DDB at 375 mg/kg. After treatment with DDB at 75 or 375 mg/kg or DDB combined with silymarin, the development of liver necrosis and fibrosis caused by CCl_4_ was markedly reduced, while after DDB combined with silymarin no DNA aneuploid cells could be observed. The decrease in glycogen and protein contents in hepatocytes caused by CCl_4_ was markedly prevented by co-treatment with DDB at 75 or 375 mg/kg or DDB combined with silymarin. It is concluded that in the model of hepatic injury caused by chronic administration of CCl_4_ in rats, the synthetic compound DDB, limits hepatocellular injury and exerts antifibrotic effect. Better improvement in protein, DNA, mucopolysaccharide content was seen after both DDB and silymarin compared to DDB alone. It is suggested, therefore, that DDB alone or in combination with silymarin might prove of benefit in the therapy of chronic liver disease. Monitoring of kidney functions in patients taking DDB is warranted.

### INTRODUCTION

Hepatitis C virus infection is the most frequent cause of chronic liver disease and the most common indication for liver transplantation. The disease is characterized by continuing necroinflammatory process and progressive deposition of extracellular matrix (fibrosis) ultimately resulting in severe architectural distortion (cirrhosis). The result is a progressive loss of liver function and end-stage liver disease necessitating liver transplantation[1,2,3]. Cirrhosis is also a risk factor for developing hepatocellular carcinoma[4]. Treatments that could halt or diminish the progression of fibrosis would thus theoretically be beneficial. Combination therapy with PEG-interferon and ribavirin has emerged as the best current treatment for chronic hepatitis C (HCV) infection, but the overall sustained response rate to PEG interferon with ribavirin is about 55%[5]. This therapy however is not feasible in a significant proportion of patients and is associated with important side effects requiring discontinuation of therapy in many instances[6,7]. Therefore, there is an immense need for developing new drugs or finding alternative strategies to prevent fibrosis and slow down disease progression.

Dimethyl-4,4'-dimethoxy-5,6,5',6-dimethylenedioxybiphenyl-2,2'dicarboxylate(dimethyl bicarboxylate; DDB) is a synthetic compound which is in use in China and other countries for the treatment of chronic hepatic diseases e.g., that caused by hepatitis C virus. The drug is a synthetic analogue of Schizandrin C, a component isolated from Fructus Schizandrae, a traditional Chinese medicine that has been shown at the beginning of 70's to lower the elevated serum alanine aminotransferase (ALT) levels of patients suffering from chronic viral hepatitis[8,9]. The reduction in serum ALT levels by DDB is considered to represent a hepatoprotective effect, since serum levels of this enzyme are sensitive indicators of liver-cell injury and are released into the blood in increasing amounts when the liver cell membrane is damaged[10,11]. Indeed rapid decrease and even normalization of serum transaminases has been noted in patients with chronic hepatitis C and B and remained normal during treatment with DDB[12,13]. Furthermore, in experimental models of hepatocyte injury, certain components isolated from Fructus Schizandrae as well as DDB decreased serum ALT levels along with an inhibitory effect on $CCl_4$-induced lipid peroxidation[8,14,15]. Other researchers, however, noted no beneficial effect on the histological grade and stage of liver disease and suggested that DDB affects the synthesis and/or degradation of ALT in liver cells and that the normalization of ALT during DDB treatment does not indicate therapeutic efficacy[13].

Silymarin (Milk Thistle, Silybum marianum) is a commonly used herbal therapy, particularly by patients who have liver disease e.g., that caused by alcohol or hepatitis C virus, where it resulted in reducing serum bilirubin, and transaminases[16,17,18]. In experimental models of hepatic injury e.g., carbon tetrachloride[19], paracetamol[20], d-galactosamine[21], ischaemia/reperfusion[22], silymarin exerted protective effects and reduced liver fibrosis induced by carbon tetrachloride[23], bile-duct ligation[24] or ethanol[25]. This hepatoprotective effect of silymarin has been suggested to result from membrane-stabilizing, free radicals scavenging properties[19], inhibition of lipid peroxidation[26] and modulation of hepatocyte $Ca^{++}$[27].

The ability of both DDB and silymarin to reduce serum transaminases and hepatocyte injury has prompted the development of commercial preparations containing mixture of silymarin and DDB for the treatment of chronic liver disease. Therefore the present study was designed to investigate the effect of DDB alone or in combination with silymarin on acute hepatic damage caused by the administration of carbon tetrachloride in rats. It was also aimed to see whether combined therapy with silymarin and DDB would offer any potential advantage over therapy with either drug alone. The effect of both drugs was evaluated on biochemical markers, histologically as well as by histochemical techniques. The severity of liver fibrosis and DNA content in hepatocytes were determined by image analysis system.

### MATERIALS AND METHODS

#### *Animals*

Sprague-Dawley rats, weighing 130-150g of body weight were used. They were housed under standard laboratory conditions with free access to standard laboratory chow and water. All animal procedures were performed according to approved protocols and in accordance with the recommendations for the proper care and use of laboratory animals.

#### Drugs and Chemicals

Carbon tetrachloride (BDH Chemicals, England), biphenyldimethyldicarboxylate (DDB), silymarin (SEDICO, Cairo) were used in the experiments. Biphenyldimethyldicarboxylate and silymarin were dissolved in saline to obtain the necessary doses. The doses of DDB and silymarin used in the study were chosen based on other studies in which the drugs produced beneficial effects in models of hepatic injury[14,15,28,29].

#### The Carbon Tetrachloride Model of Hepatic Damage

Liver damage was induced by $CCl_4$-olive oil given orally (1:1, 2.8 ml/kg). One week after the initial dose of $CCl_4$, rats were administered $CCl_4$ (1.4 ml/kg) weekly so as to maintain hepatic damage. Starting on the time of the first dose of $CCl_4$ administration, rats (n = 6/group) also received either DDB at four dose levels (3, 15, 75 or 375 mg/kg), silymarin (22 mg/kg) or a combination of DDB (75 mg/kg) and silymarin (22 mg/kg) orally daily for 30 days. Control rats were treated with olive oil (2.8 ml/kg followed by 1.4 ml/kg one week later) (n = 6). One more group (n = 6 each) received saline, but no $CCl_4$ daily for 30 days. The animals were killed 30 days after the first dose of $CCl_4$ or olive oil administration. Rats had free access to food and drinking water during the study.

#### Biochemical Analyses

Blood samples obtained from the retro-orbital vein plexuses under ether anaesthesia were analyzed for alanine aminotransferase (ALT), aspartate aminotransferase (AST), alkaline phosphatase (ALP), serum bilirubin, urea, creatinine, total proteins and glucose levels. ALT and AST activities in serum were measured according to Reitman-Frankel colorimetric transaminase procedure[30], whereas colorimetric determination of ALP activity was done according to the method of Belfield and Goldberg[31], using commercially available kits (BioMérieux, France).

#### Histological and Histochemical Studies

After the end of the treatment period, rats were killed; livers of the different groups were excised and fixed in 10% formalin saline. Paraffin sections 5 μ thick were prepared and stained with haematoxylin and eosin (H & E) for the histological investigations. Further sections were stained by Periodic acid-Schiff stain and bromophenol for intracellular mucoploysaccharides and protein detection, respectively.

#### Quantitative Measurements

Quantitative measurement of the severity of liver fibrosis and DNA content were achieved by using computerized image analyzer (Qwin Leica image processing and analysis system, Cambridge- England). To measure the severity of liver fibrosis, Masson's trichrome stain stained sections were used. The image analyzer was first calibrated automatically to convert the measurement units (Pixels) produced by image analyzer program into actual micrometer units. Ten fields were chosen in each specimen and the mean values were obtained. DNA content analysis was performed on sections stained with Feulgen methods. For each section 100-120 cells were randomly measured. The threshold values were defined by measuring control cells. The result is presented as histogram, in which normal diploid cells are separated clearly from aneuploid cells.

#### Statistical Analysis

All results are expressed as means ± SE. Comparison of the values before and after $CCl_4$ was made by paired Student's t-test. Multiple group comparisons were performed by ANOVA followed by Duncan test. $P < 0.05$ was considered statistically significant.

## RESULTS

### $CCl_4$-Induced Hepatic Injury

### Biochemical Results

The administration of $CCl_4$ was associated with marked increase in serum alanine aminotransferase (ALT), aspartate aminotransferase (AST), alkaline phosphatase (ALP) and bilirubin by 92.8%, 104.6%, 55% and 208%, respectively (Table 1). The administration of DDB in $CCl_4$-treated rats at 3 or 15 mg/kg failed to reduce the indices of hepatocellular injury, while DDB at higher doses of 75 or 375 mg/kg resulted in 61.2-76.2% decrease in ALT and 46.9-60.8% decrease in AST, respectively compared with the $CCl_4$ control group. Serum ALP did not change after DDB (15, 75 or 375 mg/kg) treatment. Silymarin (22 mg/kg) treatment resulted in 34.6, 30 and 36% decrease in ALT, AST and ALP, respectively. Meanwhile, DDB (75 mg/kg) combined with silymarin (22 mg/kg) resulted in 58.2, 31 and 19.4% decrease in ALT, AST and ALP, respectively. The increase in serum bilirubin in $CCl_4$-treated rats was not altered by either treatment. Serum creatinine increased by 32% in $CCl_4$-treated rats compared with the vehicle-treated group. In the $CCl_4$-treated rats, serum creatinine was reduced to near normal value by silymarin, but increased by further 50% after the highest dose of DDB. Serum glucose increased by 41% in the $CCl_4$-treated control group and restored to near normal values after silymarin or DDB treatment. Serum proteins did not change in $CCl_4$-treated rats.

**Table 1.**
**Serum bilirubin, urea, creatinine, proteins, glucose and enzyme activities of alanine aminotransferase (ALT), aspartate aminotransferase (AST) and alkaline phosphatase (ALP) in $CCl_4$-treated rats treated with biphenyldimethyl-dicarboxylate (DDB) and/or silymarin**

| | ALT (U/l) | AST (U/l) | ALP (U/l) | Total bilirubin (mg/dl) | Urea (mg/dl) | Creatinine (mg/dl) | Total protein (g/l) | Glucose (mg/dl) |
|---|---|---|---|---|---|---|---|---|
| Saline (normal) | 34.5 ± 3.4 | 58.5 ± 5.7 | 90.11 ± 8.0 | 0.50 ± 0.03 | 59.27 ± 3.96 | 0.50 ± 0.06 | 11.24 ± 0.66 | 59.27 ± 3.96 |
| $CCl_4$ (control) | 66.5 ± 5.7$^{\#}$ | 119.7 ± 6.6$^{\#}$ | 139.7 ± 4.3$^{+}$ | 1.54 ± 0.12$^{\#}$ | 48.19 ± 3.5 | 0.66 ± 0.06 | 11.42 ± 0.5 | 83.85 ± 6.5$^{\#}$ |
| $CCl_4$+ silymarin 22 mg/kg | 43.5 ± 6.8$^{*}$ | 83.8 ± 5.6$^{*}$ | 89.3 ± 2.7$^{*}$ | 1.41 ± 0.14 | 67.55 ± 3.4 | 0.53 ± 0.08 | 11.53 ± 0.26 | 57.39 ± 5.7$^{*}$ |
| $CCl_4$ + DDB | | | | | | | | |
| 3 mg/kg | 68.6 ± 4.6 | 104.7 ± 8.3 | 90.5 ± 2.6$^{*}$ | 1.44 ± 0.13 | 60.98 ± 7.0 | 0.64 ± 0.02 | 12.24 ± 0.42 | 61.22 ± 4.4$^{*}$ |
| 15 mg/kg | 56.8 ± 3.9 | 101.4 ± 6.4 | 123.2 ± 8.2 | 1.34 ± 0.16 | 65.13 ± 3.8 | 0.74 ± 0.06 | 12.25 ± 0.37 | 60.73 ± 4.3$^{*}$ |
| 75 mg/kg | 25.8 ± 2.8$^{*}$ | 74.2 ± 4.3$^{*}$ | 114.0 ± 8.4 | 1.66 ± 0.2 | 67.49 ± 2.4 | 0.76 ± 0.01 | 11.76 ± 0.79 | 55.22 ± 4.6$^{*}$ |
| 375 mg/kg | 15.8 ± 1.7$^{*}$ | 54.8 ± 5.3$^{*}$ | 117.5 ± 7.5 | 1.67 ± 0.11 | 67.42 ± 1.2 | 0.99 ± 0.05$^{*}$ | 14.54 ± 0.89 | 62.74 ± 6.1$^{*}$ |
| $CCl_4$ + DDB 75 mg/kg + silymarin | 27.75 ± 3.0$^{*}$ | 82.5 ± 6.3$^{*}$ | 112.6 ± .9.4 | 1.51 ± 0.16 | 50.1 ± 3.9 | 0.65 ± 0.06 | 13.35 ± 0.98 | 41.87 ± 4.4$^{*}$ |

Results are mean ± S.E. # : P< 0.05 *vs* saline group. * : P< 0.05 *vs* $CCl_4$ control group. One way ANOVA and Duncan's multiple range test.

### Histological Results

The liver of saline-treated normal rats revealed the normal characteristic hepatic architecture (Fig. 1A). No pathological changes could be observed in the liver of rats treated with either DDB or silymarin per se. The livers of rats treated with $CCl_4$ showed cirrhotic nodules and marked fibrosis. The degree of fibrosis varied from moderate to severe. The microscopic examination of the cirrhotic nodules showed marked fibrosis, architecture distortion and the appearance of many pseudo lobules. The fibrous tissues ran in septa between the nodules (Fig. 1B). In rats treated with $CCl_4$ together with silymarin, some protection was observed in the form of diminution of cirrhosis, while moderate fibrosis, vacuolar degeneration and haemorrhagic areas were also seen. Signs of degeneration in the form of some pyknotic nuclei and karyolysis were present (Fig. 1C). Examination of liver sections from rats treated with $CCl_4$ together with DDB at 3 mg/kg revealed no protective effects as compared to the group treated with $CCl_4$-olive oil only. The livers still suffered from marked fibrosis, architecture distortion and the appearance of many pseudo lobules (Fig. 1D). Meanwhile, liver sections of rats treated with $CCl_4$ + DDB at 75 mg/kg showed mild fibrosis, vacuolar degeneration and fatty changes. Signs of degeneration in the form of pyknosis, karyolysis and karyorrhexis were seen. Red blood cells could be noticed in dilated blood sinusoids (Fig. 2A). With the higher dose of DDB of 375 mg/kg, there was minimal fibrosis, macro and micro-vacuolar degeneration and fatty changes. Signs of degeneration in the form of pyknosis, karyolysis were still seen (Fig. 2B). The administration of DDB (75 mg/kg) combined with silymarin to $CCl_4$-treated rats resulted in some protection compared with treated with $CCl_4$ only. Cirrhosis and fibrosis were decreased, while vacuolar degeneration, fatty change, signs of degeneration in the form of pyknosis, karyolysis were still seen. Hypertrophy of Kupffer cells could be noticed (Fig. 2C).

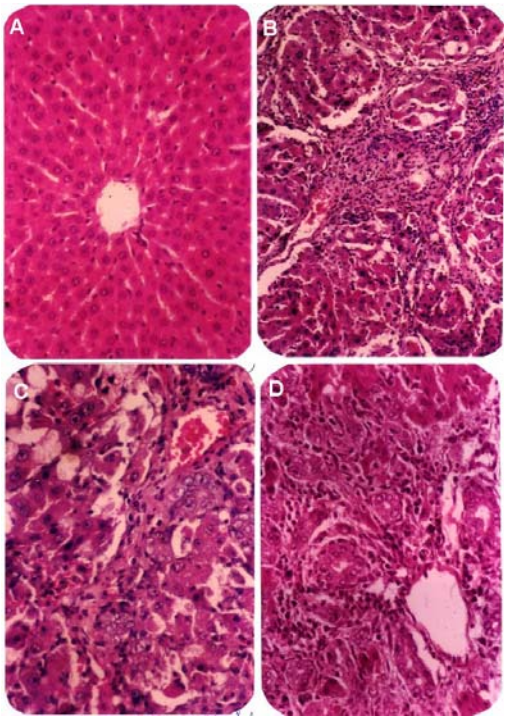

**FIGURE 1.** Section of liver from (A) an untreated control (normal) rat showing normal histological structure of hepatic lobules and central veins; (B) $CCl_4$-treated rat showing marked fibrosis (arrow), the fibrous tissue runs in septa between the nodules. Many pesudolobules could be noticed; (C) $CCl_4$ and silymarin-treated rat showing moderate fibrosis, vacuolar degeneration, haemorrhagic area, but no cirrhosis; (D) $CCl_4$ and biphenyldimethyldicarboxylate (DDB) (3 mg/kg)-treated rat showing marked fibrosis and cirrhosis (H & E x 200).

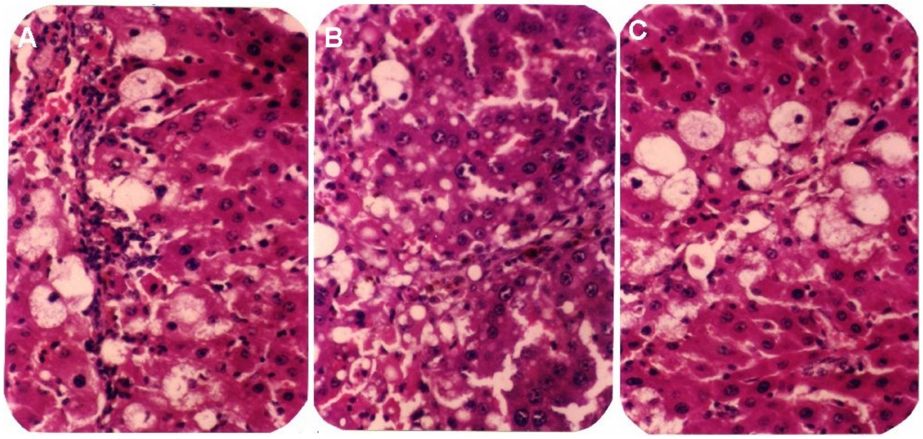

**FIGURE 2.** Section of liver from (A) $CCl_4$ and biphenyldimethyldicarboxylate (DDB) (75 mg/kg)-treated rat showing mild fibrosis, vacuolar degeneration, some pyknotic nuclei, karyolysis and karyorrhexis; (B) $CCl_4$ and biphenyldimethyldicarboxylate (DDB) (375 mg/kg)-treated rat showing minimal fibrosis, macro and micovacuolar degeneration, some pyknotic nuclei and karyolysis; (C) $CCl_4$ and DDB (75 mg/kg) + silymarin (22 mg/kg)-treated rat showing vacuolar degeneration, signs of degeneration in the form of pyknosis, karyolysis and hypertrophy of Kupffer cells (H & E x 200).

### *Image Analysis of Liver Fibrosis*

Examination Masson's trichrome stained sections from normal rats showed that collagen occurred as wavy fibril either singly or fused together in dense bundles (Fig. 3A). Liver fibrosis was assessed directly by the hepatic morphometric analysis that has been considered the gold standard for quantification of fibrosis. Significant increase in the area of fibrosis ($p<0.05$) was observed in liver sections from rats treated with $CCl_4$ only or $CCl_4$ together with DDB at 3 mg/kg (Table 2 & Fig. 3B). Significant decrease in the area of fibrosis was seen in liver sections from rats treated with $CCl_4$ together with DDB at 15, 75 or 375 mg/kg compared with $CCl_4$ control group (Fig. 3C). Significant decrease in the area of fibrosis was also observed after treatment with DDB (75 mg/kg) combined with silymarin (Table 2 & Fig. 3D).

**Table 2.**
**Quantitative study of the area of fibrosis in different groups**

| Treatment | Area of damage |
|---|---|
| Saline (normal) | $0.0 \pm 0.0$ |
| $CCl_4$ control | $42.2 \pm 5.2$ |
| + silymarin 22 mg/kg | $20.2 \pm 5.2^*$ |
| + DDB 3 mg/kg | $38 \pm 4.1$ |
| + DDB 15 mg/kg | $26.9 \pm 4.0^*$ |
| + DDB 75 mg/kg | $23.6 \pm 2.3^*$ |
| + DDB 375 mg/kg | $22.0 \pm 3.1^*$ |
| + DDB 75 mg/kg + silymarin 22 mg/kg | $21.9 \pm 1.4^*$ |

*P*< 0.05 *vs* $CCl_4$ control group. One way ANOVA and Duncan's multiple range test.

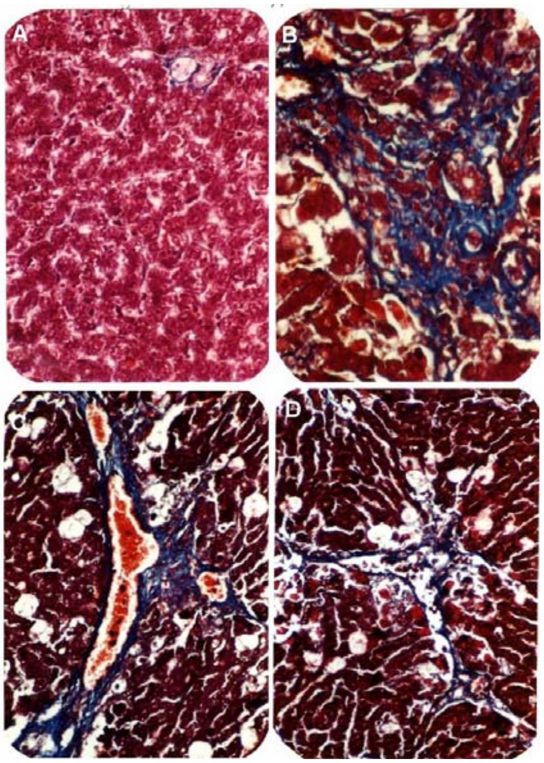

**FIGURE 3.** Section of liver stained with Masson trichrome stain showing collagen occurred as wavy fibrils either singly or fused together in dense bundles (A) control (normal) rat; (B) $CCl_4$-treated rat: increased deposition of fibrous tissue; (C) $CCl_4$ + biphenyldimethyldicarboxylate (DDB) (375 mg/kg)-treated rat: moderate amount of fibrous tissue; (D) $CCl_4$ + DDB (75 mg/kg) + silymarin (22 mg/kg)-treated rat: mild deposition of fibrous tissue (Masson trichrome stain x 200).

**Table 3.**
**DNA content in rat hepatocytes treated with $CCl_4$, silymarin, DDB or DDB + silymarin**

| | <1.5c | | 1.5c-2.5c | | 2.5c-3.5c | | 3.5c-4.5c | | >4.5c | |
|---|---|---|---|---|---|---|---|---|---|---|
| Group | % cells | DNA index | | DNA index | | DNA index | | DNA index | | DNA index |
| Saline (normal) | 22.0 ± 0.15 | 0.65 | 71.0 ± 0.26 | 1.01 | 14.0 ± 0.21 | 1.37 | 2.0 ± 0.63 | (2.02) | 0.00 | |
| $CCl_4$ control | 8.0 ± 0.21 | 0.63 | 60.0 ± 0.28 | 1.03 | 29.0 ± 0.33 | 1.46 | 2.0 ± 0.25 | (1.86) | 3.0 ± 0.4 | 2.66 |
| + DDB 3 mg/kg | 3.0 ± 0.2 | 0.56 | 31.0 ± 0.29 | 1.04 | 41.0 ± 0.31 | 1.49 | 20.0 ± 0.26 | (1.95) | 5.0 ± 0.6 | 2.87 |
| + DDB 75 mg/kg | 22.0 ± 0.22 | 0.65 | 38.0 ± 0.29 | 1.01 | 31.0 ± 0.25 | 1.45 | 10.0 ± 0.22 | (1.94) | 2.0 ± 0.04 | 2.48 |
| + DDB 375 mg/kg | 16.0 ± 0.1 | 0.68 | 52.0 ± 0.28 | 1.01 | 30.0 ± 0.23 | 1.41 | 6.0 ± 0.12 | (1.87) | 2.0 ± 0.01 | 2.33 |
| + DDB 75 mg/kg + silymarin 22 mg/kg | 66.0 ± 0.18 | 0.59 | 34.0 ± 0.2 | 0.86 | 0.00 | | 0.00 | | 0.00 | |

* *P*< 0.05 (ANOVA) *vs* $CCl_4$ control group. One-way ANOVA and Duncan's multiple range test.

### Histochemical Results

### DNA Content

Image analyzer automatically expresses the DNA content of each individual cell measured then give the percentage of each cell out of the total number of cells examined and classifies cells into four categories namely diploid (2c), proliferation index (s-phase cells)(3c), tetraploid (4c) and cells with more than 4.5c DNA content which indicates aneuploidy. Table 3 & Fig. 4A shows the DNA content in normal rats. The liver of rats treated with $CCl_4$ only resulted in 3% of the examined cells more than 4.5c (aneuploid), 60% of cells contained diploid DNA value (2c), 29% of the examined cells contained 3c DNA value (proliferation index was high), 2% of the examined cells were at the 4c area (tetraploid), 18% of the examined cells contained DNA<1.5c (Table 3 & Fig. 4B). Examination of liver sections from rats treated with $CCl_4$ together with DDB at 15 mg/kg showed that 5% of the examined cells were more than 5c (aneuploid), 31% of the cells contained diploid DNA value (2c), 41% contained 3c DNA (proliferation index was high), 20% were at the 4c area (tetraploid) (Table 3). In liver sections from rats treated with $CCl_4$ and DDB at 75 mg/kg, 38% of cells contained diploid DNA value (2c), 31% of cells contained 3c DNA value (high proliferation index), 10% of cells were at 4C area (tetraploid) (Table 3). In liver sections from rats treated with $CCl_4$ and DDB at 375 mg/kg, 52% of cells contained diploid DNA value, 16% of cells contained DNA <1.5c, 30% contained 3c DNA value (high proliferation index) (Table 3 & Fig. 4C). In liver sections from rats treated with $CCl_4$ and DDB combined with silymarin, 34% of cells contained diploid DNA value, 66% of cells contained DNA <1.5c, no DNA aneuploid cells could be observed proliferation index equal zero (Table 3 & Fig. 4D).

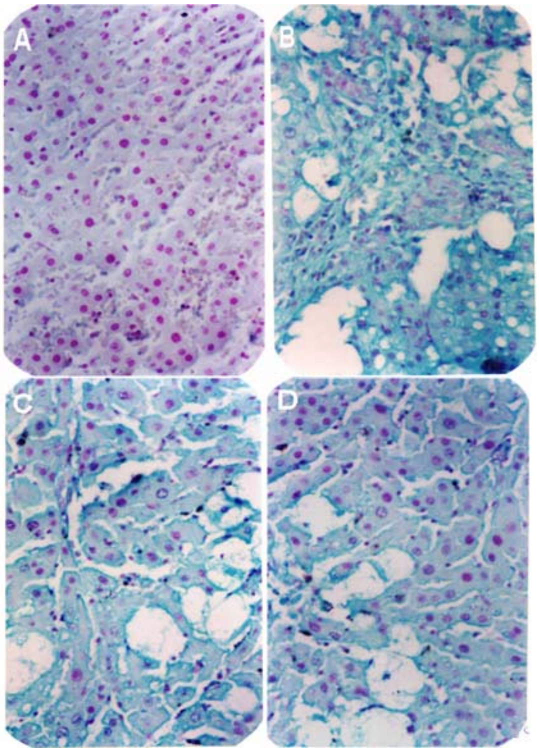

**FIGURE 4.** Section of liver stained with Feulgen stain showing DNA in hepatocytes (A) control (normal) rat; (B) $CCl_4$-treated rat showing decreased DNA contents; (C) $CCl_4$ + DDB (375 mg/kg)-treated rat showing mild improvement in DNA content; (D) $CCl_4$ + DDB (75 mg/kg) + silymarin (22 mg/kg)-treated rat showing moderate improvement in DNA contents (Feulgen reaction x 200).

### Glycogen Content

Fig. 5A shows the distribution of glycogen contents in liver tissue stained by Periodic acid Schiff's (PAS), characterized in normal rats by deeply stained reddish granules in the cytoplasm of hepatocytes. The peripheral zonal cells showed higher mucopolysaccharide content than the central cells. The glycogen content was sharply reduced in the cytoplasm of hepatocytes from rats treated with $CCl_4$ alone (control) or $CCl_4$ combined with DDB 15 mg/kg (Fig. 5B). In rats treated with $CCl_4$ combined with DDB 75 or 375 mg/kg, moderate increase in mucopolysaccharide content was observed especially in the center of the lobules (Fig. 5C), while in rats treated with $CCl_4$ combined with DDB and silymarin, mucopolysaccharide content almost approximated normal control (Fig. 5D).

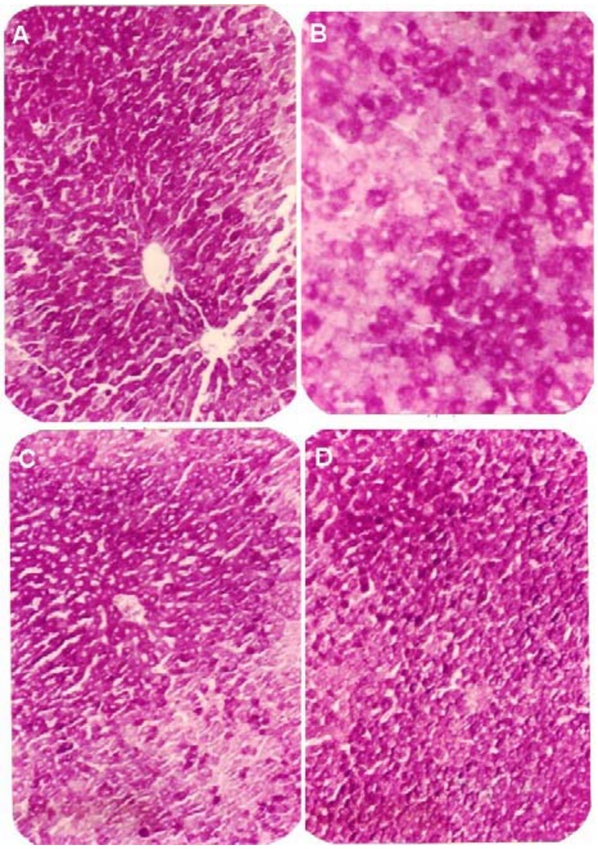

**FIGURE 5.** Section of liver stained with Periodic acid-Schiff stain showing mucopolysaccharide materials in the cytoplasm of hepatocytes: (A) control (normal) rat; (B) $CCl_4$-treated rat showing marked diminution of mucopolysaccharides in the cytoplasm of some hepatocytes; (C) $CCl_4$ + DDB (375 mg/kg)-treated rat showing increase in mucopolysaccharide content; (D) $CCl_4$ + DDB (75 mg/kg) + silymarin (22 mg/kg)-treated rat showing mucopolysaccharide contents more or less normal (Periodic acid-Schiff reaction x 200).

### Protein Content

Mercuric bromophenol blue staining was used to demonstrate total protein content in liver sections. Examination of liver sections from normal rats showed moderate protein content in the cytoplasm of hepatocytes. Some nuclei showed deep protein content (Fig. 6A). Rats treated with $CCl_4$ alone (control) or $CCl_4$ combined with DDB 15 mg/kg exhibited marked diminution of protein content in the cytoplasm of hepatocytes and the stainability was mostly diffused (Fig. 6B). Moderate increase in protein content was recorded in case of rats with $CCl_4$ along with DDB 75 or 375 mg/kg, compared to rats treated with

$CCl_4$ alone (Fig. 6C). An increase in protein content was also observed in case of rats with $CCl_4$ along with DDB and silymarin, compared to rats treated with $CCl_4$ alone (Fig. 6D).

### DISCUSSION

The present study provides evidence that in a model of hepatic injury and fibrosis caused by chronic administration of $CCl_4$ in rats, the synthetic compound DDB decreased leakage of hepatocellular enzymes ALT and AST into plasma and lessened the development of hepatic fibrosis necrosis and fibrosis caused by $CCl_4$. Cellular dysfunction induced by $CCl_4$ and evidenced by reduced glycogen and protein content in hepatocytes and DNA alterations was largely prevented by DDB**.** Similar findings were observed on treatment with silymarin. These findings indicate that both DDB and silymarin limit hepatocellular injury and exert antifibrotic effect. A benefit from combining both DDB and silymarin was seen on histochemical investigation in the form of a better improvement in protein, DNA, mucopolysaccharide content compared to DDB alone.

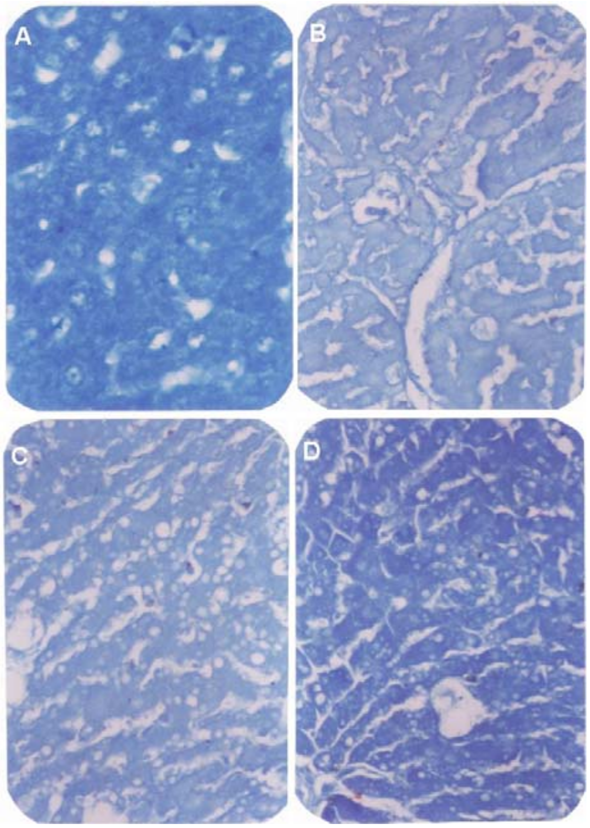

**FIGURE 6.** Section of liver stained with bromophenol blue showing greenish blue protein content in the cytoplasm of hepatocytes: (A) control (normal) rat; (B) $CCl_4$-treated rat showing marked diminution of protein content; (C) $CCl_4$ + DDB (375 mg/kg)-treated rat showing moderate improvement in DNA content; (D) $CCl_4$ + DDB (75 mg/kg) + silymarin (22 mg/kg)-treated rat showing that protein content approximated control (Bromophenol blue stain x 200).

In a number of *in vivo* and *in vitro* models of hepatocyte injury, the administration of DDB has been reported to protect the liver from a variety of toxicants. The drug decreased serum ALT levels along with an inhibitory effect on $CCl_4$-induced lipid peroxidation and on the binding of $CCl_4$-metabolites to lipids of liver microsomes[8,14,15,32]. In isolated suspended rat hepatocytes, DDB protected the hepatocytes against $CCl_4$ and D-galactosamine induced damage. Membrane lipid peroxidation (malondialdehyde formation) and ALT release from the hepatocytes were markedly decreased. The damage of the cell surfaces of the hepatocytes was also reduced as seen under a scanning electron microscope[32]. Increased oxidative stress is a feature of $CCl_4$-induced liver injury in which Kupffer cells and neutrophils have significant role[33,34]. Excessive lipid peroxidation results from $CCl_4$ due to formation of trichloromethyl free radical within the membrane of the endoplasmic reticulum[35,36]. The beneficial effects of DDB in protecting the hepatocytes from $CCl_4$-induced damage could thus be the result of decreased oxidative burden. In other studies, DDB reduced hepatic fatty degeneration induced by ethanol treatment in rats along with an inhibitory effect on malondialdehyde formation in liver homogenates[37]. The administration of DDB also ameliorated the reduction of liver glycogen and blood glucose caused by D-galactosamine in mice[14]. Liver injury mediated by immune response in concanavalin A-treated mice was markedly reduced by prior administration of DDB with serum ALT and bilirubin being decreased[38]. In man and despite the widespread use of DDB in patients with chronic liver diseases, little information is available as regards to the effect of this drug on disease progression. In patients with chronic liver disease, rapid decrease and even normalization of serum transaminases has been reported[12,13].Unlike ALT levels, aspartate aminotransferase, gamma-glutamyl transferase and glutamate dehydrogenase levels were not affected and it was suggested that the normalization of ALT during DDB treatment does not indicate therapeutic efficacy[13].

Silymarin, a standardized plant extract, has a worldwide reputation as a hepatoprotective agent. In chronic alcoholic liver disease, silymarin reduced serum bilirubin, and transaminases[16,17]. Others, however, failed to demonstrate such benefit from silymarin in patients with chronic hepatitis C infection[11]. Studies in patients with liver disease have shown that silymarin increased superoxide dismutase activity of lymphocytes and erythrocytes[39], increased glutathione levels and decreased lipid peroxidation in peripheral blood cells[40,41].

In the present study, treatment of rats with $CCl_4$ for one month induced marked fibrosis, architectural distortion, cirrhotic nodules and the appearance of many pseudo lobules, which in accordance with other studies[42,43]. Hepatic fibrosis is the most important pathogenic mechanism towards end-stage liver disease necessitating liver transplantation[3]. Liver fibrosis represents the response of the liver to diverse chronic insults e.g., viral, toxic, metabolic and immunological. Regardless of the aetiology, liver fibrosis is characterized by an increase in extracellular matrix proteins including collagen in perisinusoidal and periportal spaces that collectively form hepatic scars. The significance of liver fibrosis derives from the fact that it progresses rapidly to cirrhosis in several clinical settings[44] and is a high risk factor for developing hepatocelluar carcinoma[4]. Hepatic inflammation appears to be the key pathological substrate that drives fibrogenesis. This makes sense as inflammation and cellular damage are intuitively linked to the fibrotic process via leucocyte/hepatic stellate cell (HSC) interaction, cytokine networks, proinflammatory small molecules (for example, reactive oxygen species), and insoluble mediators such as the hepatic neomatrix during wound healing[45].

In the present work, treatment of rats with DDB or silymarin along with $CCl_4$ resulted in diminution of cirrhosis and fibrosis. Other researchers reported that dimethylnitrosamine (DMN)-induced liver fibrogenesis, however, was not reduced by DDB[28]. In rats treated with $CCl_4$, Favari and Perez-Alvarez[46] reported a 55% reduction in liver collagen by silymarin. In rats with secondary biliary cirrhosis, silymarin reduced hepatic collagen accumulation by 35%[47]. It is worth to note that both DDB and silymarin possess important anti-inflammatory properties, which are likely to be of relevance to their hepatoprotective and antifibrotic effects. Kang et al.[28] suggested that DDB decreases liver cell injury and inflammation through inhibition of nuclear factor-kB. In an immune-mediated liver injury caused by concanavalin A in mice, the elevation of serum TNF-alpha and liver TNF-alpha mRNA expression 2 h after Con A injection was decreased by the prior administration of DDB[38]. Silymarin inhibited the migration of neutrophils into the inflamed site[48], an important early event in the inflammation cascade. The reduction of plasma levels of the liver associated enzymes, ALT and AST by DDB or silymarin observed in the present study and in other studies suggests a reduction of hepatic inflammation by either drug. Aminotransferase levels are sensitive indicators of liver-cell injury. Both enzymes are released into the blood in increasing amounts whenever the liver cell membrane is damaged. It is thus likely that the observed effect of DDB or silymarin on hepatic fibrosis in the present study is a consequence of a reduction in the inflammatory response caused by $CCl_4$.

Image cytometery for DNA quantitation has become an established technique in the field of analytical cellular pathology, providing significant information about biological behavior of cells[49]. In the hepatocyte, clonal perpetuation of accumulated DNA aneuploid affecting growth regulatory genes eventually leads to a continuation of severe progressive abnormalities characterized by dysplasia, formation of adenoma and, finally, cancer[50]. In the present study, treatment of rats with $CCl_4$, resulted in 3% of hepatocytes being aneuploid (>5c), while 60% of the examined cells contained diploid DNA value (2c) together with high proliferation index. Carbon tetrachloride-induces liver cell dysplasia which is a step towards the development of hepatocellular carcinoma. The dysplastic hepatocytes shows aneuploid DNA patterns which increase with increasing histological grade and stage of tumours[51,52]. In the present study, in liver sections from rats treated with $CCl_4$ and DDB at 75 mg/kg combined with silymarin, no aneuploid cells could be noticed, while proliferation index equaled zero. Other researchers reported a chemopreventive and tumour reversing effect for biphenyl dimethyl dicarboxylate on malignant transformation[53,54]. DDB significantly inhibited hepatocyte nuclear DNA fragmentation 12 h after Con A injection and prevented the direct DNA damage induced by CuSO *in vitro*[38]. In human prostate cancer (PCA) PC3 cells, silymarin and its principle component silibinin inhibited cell proliferation, induced cell death, and caused G1 and G2-M cell cycle arrest in a dose/time-dependent manner[55]. Silibinin treatment of human bladder cancer cells had similar effects[56].

The present study in addition aimed to investigate whether there would be a benefit from combining both DDB and silymarin. It was observed that the combination DDB 75 mg/kg + silymarin did not improve AST, ALT and ALP activity compared with DDB 75 or 375 mg/kg alone. In addition, the effect of DDB (75mg/kg) plus silymarin (22 mg/kg) on area of fibrosis was similar to that obtained with the administration of DDB alone. Nevertheless, a benefit from combining both DDB and silymarin was observed on histochemical investigation in the form of a better improvement in protein, DNA, mucoplysharrchide content compared to DDB alone. Furthermore, no aneuploid cells were present when silymarin was combined with DDB. Silymarin has metabolic and cell-regulating effects at concentrations found in clinical conditions, namely carrier-mediated regulation of cell membrane permeability, inhibition of the 5-lipoxygenase pathway, scavenging of reactive oxygen species and an action on DNA-expression, for example, via suppression of nuclear factor (NF)-kappaB[57]. This in addition to the effect of silymarin on cell proliferation[55,56] suggests that silymarin may be a useful additive therapy in patients with chronic liver disease treated with DDB.

In summary, hepatocellular injury and hepatic fibrosis evoked by the administration of $CCl_4$ for one month in rats was ameliorated by the co-administration of the synthetic compound DDB. Furthermore, these effects were associated with a reduction in aneuploid cells particularly when DDB was given together with silymarin. The drug in combination with silymarin is therefore likely to be of benefit in reducing the likelihood of malignant transformation in patients with chronic liver disease. With high doses of DDB, blood urea and creatinine were raised which warrants monitoring of kidney functions in patients taking the drug.
